# Multisensory Food Experiences in Northern Norway: An Exploratory Study

**DOI:** 10.3390/foods13132156

**Published:** 2024-07-08

**Authors:** Huy Tran, Nina Veflen, Eva J. B. Jørgensen, Carlos Velasco

**Affiliations:** 1 Centre for Multisensory Marketing, Department of Marketing, BI Norwegian Business School, 0484 Oslo, Norway; nina.veflen@bi.no (N.V.); carlos.velasco@bi.no (C.V.); 2 School of Business and Economics, UiT, The Arctic University of Norway, 9510 Alta, Norway; eva.joergensen@uit.no

**Keywords:** multisensory, food, experiences, Arctic, Sámi, Northern Norway

## Abstract

Intrinsic and extrinsic sensory elements influence our food experiences. However, most research on extrinsic multisensory aspects of food has centered on WEIRD (White, Educated, Industrialized, Rich, and Democratic) urban participants. This study breaks from this trend by investigating multisensory food experiences in the context of Northern Norway, a region characterized by distinct seasonal shifts, harsh arctic weather, unique atmospheric phenomena (e.g., the midnight sun and northern lights), limited food growth opportunities, and a rich Sámi cultural heritage. Our aim was to unravel the formation and development of multisensory food experiences within a culturally and environmentally specific framework. Our exploratory research used participant observation and interviews, involving four researchers from diverse backgrounds who closely examined multisensory food experiences within four Northern Norwegian food-related tourism businesses, all infused with Sámi cultural elements. Our findings suggest four major themes: (1) *Experience elements*, involving elements associated with plants, animals, and inanimate objects; (2) *Bipolar concepts*, which refer to opposing dimensions where experience elements varied, notably in the interplay between Sámi and Norwegian traditions; (3) *Sensory stories*, which highlight the narratives, enriching the eating experience with context, such as tales of dining under the captivating northern lights; and (4) *Values*, which indicate guiding principles shaping these experiences on a broader scale, emphasizing support for local traditions and culture. Our main contribution is the presentation of a new contextual framework of multisensory food experiences, which can be applicable to studying food experiences in other contexts.

## 1. Introduction

Food experiences are multisensory in nature, and thus, what is presented to our senses plays a key role in shaping our perception, enjoyment, and overall satisfaction with what we consume [1]. Extensive research has been devoted to the importance of both intrinsic factors (such as taste, texture, and aroma) and extrinsic factors (including visual presentation, auditory cues, and social context) in shaping these experiences (e.g., [2,3]) and how food businesses such as restaurants are utilizing these factors to design experiences [4]. However, most studies investigating the multisensory dimensions of food have been confined to urban areas, predominantly focusing on participants from the White, Educated, Industrialized, Rich, and Democratic (WEIRD) [5] demographics (but see [6] for a representative exception). This limited scope hinders our understanding of food experiences in diverse cultural and environmental contexts as well as the generalizability of previous findings in multisensory food experiences [7].

Exploring the context outside the WEIRD domain can offer a more nuanced understanding of multisensory food experiences. By employing a context-sensitive approach, we can uncover a broader range of daily practices that can deepen the insight into diverse food experiences [8,9]. Moreover, emphasizing the interplay between actors and their contexts can provide a stronger grounding of theories and concepts, a more balanced view of the relationships between agency and structure, and pave the way for approaches and methods that are more attuned to socioecological factors and context [10].

The present study focuses on exploring the multisensory food experiences in the northernmost region of Norway. By focusing on this specific context, our aim is to unravel the formation and development of multisensory food experiences within a culturally and environmentally specific framework. Examining food experiences in the northern region of Norway allows us to broaden our comprehension of the complex interplay between sensory stimuli, cultural influences, and environmental factors in shaping the perception and enjoyment of food. To achieve our objectives, we used a qualitative approach, encompassing participant observation and interviews. This approach enabled us to capture the different dimensions of multisensory food experiences while also considering the socio-cultural and environmental factors at play. Our research is mostly exploratory in nature and aims to inspire more diverse research in the context of multisensory experiences.

First, we introduce the context of the multisensory food experiences in Northern Norway and present the current literature on multisensory food experiences. Following the Methods section, our Results section shows the importance of non-immediate environmental factors for shaping food experiences. Then, we propose a contextual framework of the multisensory food experiences and discuss potential contributions of our research. 

### 1.1. The Context of Multisensory Food Experiences in Northern Norway

*Northern Norway*. This region consists of the two northernmost counties in Norway: Nordland and Troms og Finnmark. The region is rural and sparsely populated, with around 483,500 people living on nearly 113,000 km^2^ [11]. Only five cities have more than 20,000 inhabitants, and the distances between them are considerable [11]. The region has a tough arctic climate and marked seasonal changes, though this is now changing toward a warmer and wetter climate [12]. Furthermore, the region’s geographical characteristics give rise to relatively limited opportunities for food growth, fostering a reliance on local resources and traditional culinary practices as well as imports [13]. Part of Northern Norway is north of the Polar Circle, also called the Arctic Circle (66°33′) [14]. Above this latitude, there is at least a day a year when the center of the sun is continuously above the horizon (the midnight sun) and at least a day a year when it is always below the horizon (polar night) [15]. The further north, the longer the periods with polar night and midnight sun will last (e.g., at Svalbard it will last for 5 months). 

*Sámi people*. Northern Norway is part of the core area of the Sámi people. The Sámi people also live in middle and northern parts of Sweden, northern Finland, and the Kola Peninsula in Russia. There are around 75,000–100,000 Sámi people living in these four countries; of these, about 45,000 are in Norway [16]. The communities inhabiting Northern Norway possess a rich cultural heritage, with the indigenous Sámi people adding layers of history and identity to this context [17].

The Sámi community is diverse, with nine unique languages, each with its own distinct cultural and regional characteristics [18]. Due to the processes of colonialization and Norwegianization, many Sámi people do not speak the Sámi language [19,20]. Being born and raised in a Sámi reindeer herder family, and taking part in this family business, is one example of strong Sámi cultural belonging [21]. Notably, the Sámi people are the only Indigenous people in the European Union [22].

*Experiences in Northern Norway*. Sámi tourism is tightly connected to the production of experience-based products. While previous studies have investigated the importance of storytelling [23] and how motivation to travel influences the valuation of the various phases of the journey in the north [24], little investigation has been devoted to multisensory food experiences in Northern Norway. Sthapit [25], who investigated memories of gastronomic experiences in Rovaniemi, Finland, is one of the most relevant studies. It showed that tasting local products such as reindeer, salmon, and cloudberry in Rovaniemi led to strong emotions of joy and created memorable local food gastronomy experiences long after the holiday of the visitors ended. While most of the findings from this study focused on taste experiences, a few of the 16 respondents interviewed also mentioned the other sensory experiences, such as the exceptional setting (like a reindeer farm and a campsite) and their fascination for the wooden plates that the foods were served on. Although the study illustrated the uniqueness of the eating experience in the North, it focused primarily on taste experiences in the Finnish tourist destination, neglecting other sensory experiences in Northern Norway. What characterizes full multisensory experiences of Northern Norway is still not well understood.

### 1.2. The Current Research in Multisensory Food Experiences

Multisensory experiences involve the deliberate integration of sensory elements like colors, sound, touch, taste, and smell, in one or a series of events, to create specific impressions [7]. In the context of food and beverages, this approach can transform meals into experientially differentiated journeys [26]. In the framework presented by Velasco and Obrist [7], key components of multisensory experiences are described. The impression, or the perceptual, emotional, and cognitive impact of these sensory elements, is the starting point. For instance, an artistically plated dessert with aromatic spices and a delightful crunch leaves a specific (e.g., exclusive experience) and lasting impression on the diner. Sensory elements in food include, for instance, the visual appeal, sounds associated with preparation or consumption, tactile sensations, smells, and tastes (e.g., [27]). Events, or specific encounters designed to engage the senses, can be seen in the preceding stages of the meal, the sequence of courses in a meal, and the post meal (cf. [28]). The receiver, or the individual experiencing the meal, brings their own socio-cultural background, preferences, and sensory sensitivities, which influence their perception. 

The aforesaid definition of multisensory experiences puts the senses at the core of experiences. Our food experiences, in particular, are shaped by a variety of sensory cues [29], which can be categorized as either intrinsic or extrinsic [3]. Intrinsic factors pertain to the inherent qualities of the food, such as its taste, aroma, and texture [1]. Extrinsic factors, on the other hand, include elements like packaging, table settings, lighting, and background music [4], which are external to the food itself but ever-present in food experiences. Given the significant impact of these sensory elements, the practice of designing multisensory food experiences is gaining popularity in various food sectors [4,7]. 

Previous research has demonstrated that immediate eating contexts, such as restaurant atmospheres, significantly influence the eating experience [30]. However, the effects of non-immediate environmental factors, such as the surrounding nature—including both animate and inanimate objects across different seasons [31]—and unique cultural factors have not been thoroughly investigated. Furthermore, while multisensory food experiences can be intentionally crafted or arise naturally, the memorable eating moments are often naturally occurring, tied to nostalgia or specific events, rather than being deliberately designed [31]. These might suggest that the non-immediate environmental factors play a vital role in shaping our food experiences.

### 1.3. Our Aims

Considering the research gap that most studies investigating the multisensory dimensions of food have been confined to urban areas, predominantly focusing on consumers that fall within the WEIRD characterization [5], we aimed to observe, experience, and document representative multisensory food experiences in Northern Norway from the perspective of the experience designers (e.g., restaurants and businesses). The participant observation was directed toward the food experiences and their corresponding extrinsic (not specific to the food itself, but everything else) multisensory components, such as the way the foods looked (sight), sounded (hearing), smelled (ortho-nasal olfaction), and felt (touch). 

The participative observation method was useful to explore the natural and cultural elements of the food experiences. Furthermore, we aimed to understand how the interplay between Sámi and Norwegian culture shaped the unique food experiences in Northen Norway. Findings from these questions can help provide future research to explore what makes food experiences in one area different from another and how we can capitalize on these differences to enhance our experiences. For the practical contributions, we hope that our findings can be useful for those who want to promote tourism that combines food with places with unique cultures and natural environments. 

## 2. Method

*Participants.* The participants of this study consisted of a group of four businesses in Northern Norway, each chosen for its unique embodiment of the region’s multisensory food experiences. All businesses are in the tourism industry, producing and/or selling food experiences with a flavor of the Sámi culture. The first produces food by blending local traditions with innovative twists, utilizing local plant-based resources. The second curates fine dining experiences with optional accommodation and unique local activities (e.g., dog sledging), underpinned by sustainability values. The third provides serene dining experiences combined with mountain sport activities in a secluded location, highlighting the region’s authentic charms. The fourth offers typical foods of the region served in a setting filled with Sámi artifacts and surrounded by reindeers. This selection of participants was decided based on deliberation with the local researcher, who is also Sámi, to ensure the inclusion of representative cases of the Northern Norwegian food experiences. This helped us capture the interplay of tradition, innovation, and authenticity that characterizes Northern Norway’s multisensory food experiences. 

All of these businesses are localized down in the valley (near the river) or on the highlands in Vest Finnmark, an area with 38,293 inhabitants, including 3378 people living within Sámi settlement areas with 78,909 reindeer [32]. This area has two months of polar night and two months of midnight sun every year, a wide temperature range (low: −22.7 °C, high: +30 °C) in the valley and even wider in the highland (low: −35.7 °C, high: +27 °C; the coldest temperature ever reported is −51.4 °C) [33]. Excellent access to seafood (around 250,000 tons landed every year), salmon from the rivers (25,000 tons), and reindeer meat; limited agricultural area (186,000 square meters of mostly grass); and distinct seasons (some state eight seasons: early spring, late spring, early summer, late summer, early fall, late fall, early winter, and late winter) makes this area unique, different from the rest of Norway and most of the rest of the world [32].

*Design and procedure*. This research followed the method of participant observation as well as semi-structured interviews. Participant observation is a qualitative data collection method that involves the researchers immersing themselves in a particular context, actively participating, and gaining a profound understanding of the *hows* and *whys* of the behaviors that occur in said context (see [34] for an overview). This research method is useful for such an exploratory study, as it allows us to collect rich and detailed data with the possibility to immerse directly into the unique context of the food experiences.

To facilitate the observations, we developed an observation guide (see Appendix A). All researchers took notes and pictures and participated in the semi-structured interviews, which were written down before comparison within the research team (for more details about the observation approach, see [35]). 

**Figure 1:**
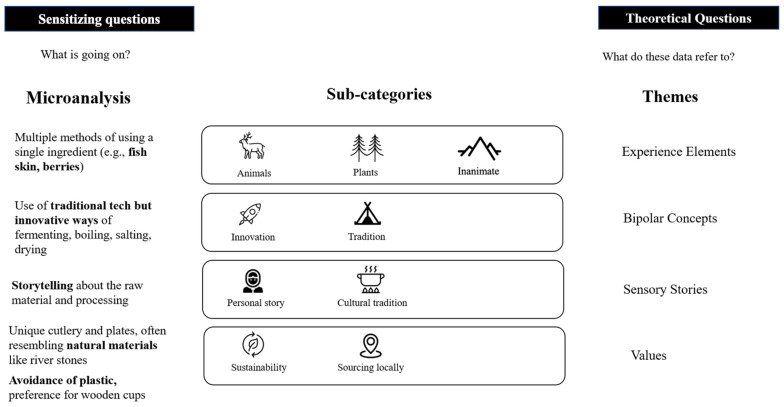
Example of the analysis. The figure is based on Laitinen et al. [35].

*Analyses.* The analyses of the participant observation data were based on grounded theory. The researchers followed the process described by Laitinen et al. [35], whereby participant observation allows researchers to understand valuable insights into what people do compared with what they say they do, and grounded theory aims to create a theory based on qualitative data, progressing through stages of open, axial, and selective coding (see Figure 1). After that, the researchers went through an iterative process of refining the establishment of concepts and categories. The researchers did not share their field notes before all observations were done to ensure that one researcher’s perspective would not influence another’s. After investigating all the notes, four themes with sub-categories emerged.

The iterative process developed by the researchers followed the definition of Velasco and Obrist of multisensory experiences, that is, “…impressions formed by specific events, whose sensory elements have been carefully crafted by someone” [7]. Here, we focused on identifying the contextual sensory elements that the business owners used to craft the food experiences. We organized them as a function of various categories of analysis. These categories of analysis consisted of the following: (1) Experience elements refer to where the sensory characteristics originate (plants, animals, inanimate objects). (2) Bipolar concepts refer to a series of contradicting dimensions along which the experience elements appear to vary, for example, along the Sámi–Norwegian traditions, in which the sensory elements group. (3) Sensory stories refer to the narratives associated with the eating experience and the way in which meaning is made out of both experience elements and concepts. For example, every time a meal or a product was presented, it came with a story (e.g., “I tried it first in a winter night with bright northern lights”). (4) Values refer to a series of principles that appear to guide the way in which the experiences form at a broader level, for example, favoring the local, simple, and sustainable (broadly understood). 

## 3. Results: Multisensory Contextual Elements Associated with the Food Experiences

**Figure 2:**
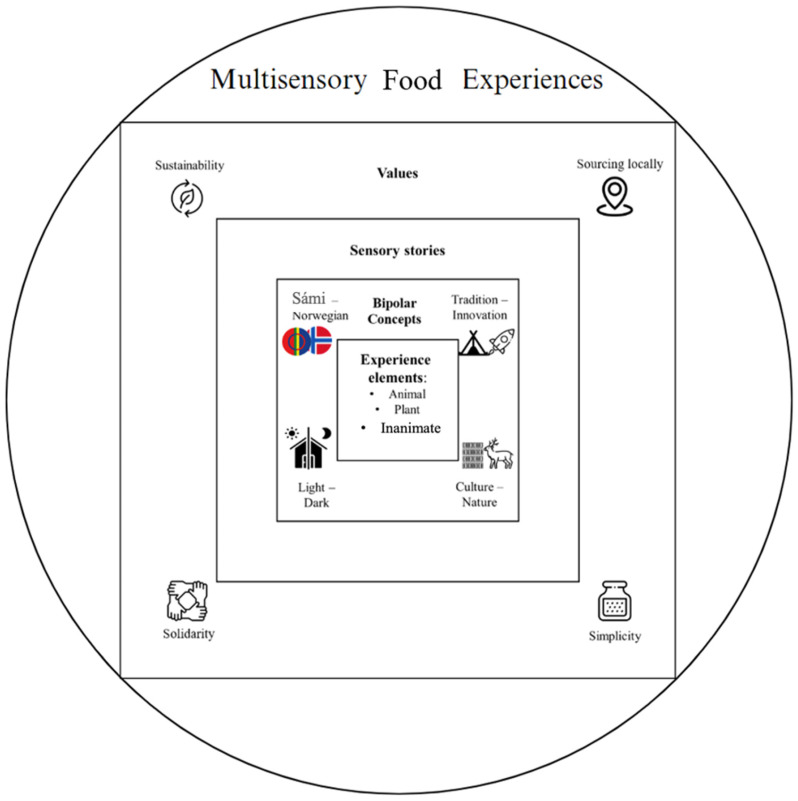
A contextual framework of the multisensory food experience in the north of Norway.

Figure 2 offers a visual representation of the context for the multisensory food experience of Northern Norway that emerged from our observations. Following the display of Figure 2, we present the result associated with each category of analysis.

*Experience elements*. These are the building blocks of sensory experiences, representing the sources from which sensory characteristics originate [7]. These elements, as captured in our observations, can be categorized into three broad categories: plants, animals, and inanimate objects (see [36] for insights into the organization of semantic associations between the senses). 

**Figure 3:**
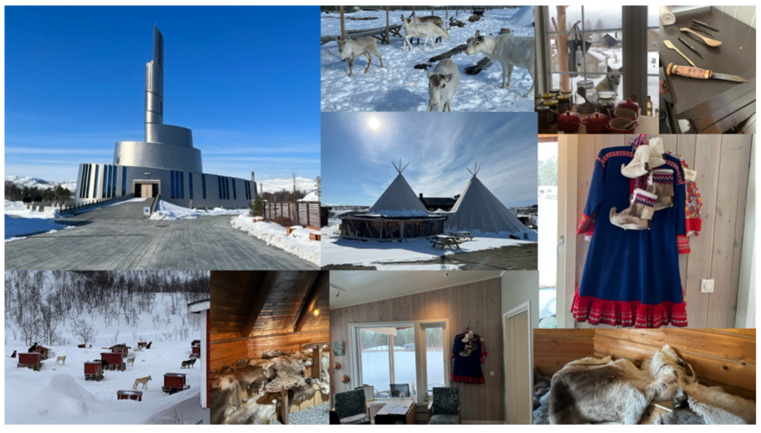
An example collage of photos taken during the research.

Experience elements originate from typical plants such as trees (birch), berries (cloudberries), and flowers (sweet meadow). These contribute intrinsic attributes to the experience such as flavors and aromas, but also extrinsic elements such as textures, colors, shapes, and smells attributed to the overall sensory experience. For example, wood is ever present in both the construction materials of the different environments in which the experiences occur (which are also highlighted in the sounds that are derived from walking on or interacting with the material) and in the sort of tools and utensils that are in them. Moreover, there is rich imagery associated with the colors, shapes, and overall visual landscape of the plants, herbs, and flowers that can be obtained in that region of the arctic. Experience elements that are derived from animals, including reindeer, seafood, and grouse, contribute not only to the flavors and aromas, given that many dishes are made from these raw materials; but also, to the textures, smells, and visual aspects associated with animals from the region. Indeed, many utensils and decorations were made from reindeer or used reindeer-based imagery. Experience elements originate also from inanimate objects such as slate (used for plates), iron (used for knives), daylight, outdoor temperatures, and other non-living components. These elements can contribute to the overall atmosphere of the multisensory experience. For instance, imagery associated with the winter and the northern lights become a key atmospheric cue and an experience by itself. See Figure 3 for an example.

Here are example reports from the researchers’ notes:

“After walking in the snow for some time, feeling the cold but fresh environment, and the vast and open space full of snow, bright and contrasted with the red wooden cabins, we entered the wooden house. The sound and smell of burning wood is characteristic. The cabin involves much wood material. The sound of the inside and outside merges. The outside involves sounds of cleaning snow from shoes and the entrance, the inside sounds involve crackling wood, voices of chatting people, as well as any interactions with the wood, or later the utensils used to eat. The dining setting minimalistic in a way, but consistently cozy. White plates with textured lines for the main, a more decorated plate for the bread, and later the waffles, and a glass for the drink. Other than the aromas of the Bidos, or later the jams, there’s not much more. The cutlery is metal-based and relatively minimalistic”.

“Around the building there where three lavvos and a fenced area for the reindeers. Two reindeers were outside the fence on a lease. These two were found lost and where to be picked up by the owners. Inside we saw some one-year-old (very hungry, could eat reinlav all day if they were allowed), 4–5 simpler (pregnant reindeer, probably delivering one calf in May (rarely they get twins, most often only one). After the mating period, the males lose their antlers. Then they cannot fight and ends up at the bottom of the hierarchy. The females still have theirs and can protect themselves and the unborn calves. At some point they will lose theirs too”.

*Bipolar concepts.* Bipolar concepts in the context of multisensory experiences refer to opposing dimensions along which sensory elements can vary (e.g., resembling semantic differentials, though context-specific differences, see [37]), which are relevant to the context of the different businesses. These dimensions represent contrasting attributes or characteristics that can be used to describe and differentiate sensory experiences. Here, we identified four bipolar dimensions along which the different experience elements can be organized, namely, (1) Sámi vs. Norwegian, (2) tradition vs. innovation, (3) light vs. dark, (4) culture vs. nature. 

The Sámi vs. Norwegian bipolar dimension represents the contrast between the parts of the experience that are associated with Sámi and Norwegian traditions. The Sámi component may exhibit distinct sensory attributes influenced by Indigenous traditions (e.g., reindeer as a feast meat for a big wedding with more than 1000 attendees) and the natural surroundings of the Arctic region (e.g., diet mainly consisting of protein, not vegetables, until the 1700s). In contrast, the Norwegian component may reflect different sensory attributes influenced by the general tendencies in Norway (e.g., coffee after meals and desserts with waffles and jam). In all the observations, while there was a tension, there was also a sort of interest in maintaining local authenticity while acknowledging that the broad Norwegian culture was also part of it.

“We talked in Norwegian, while drinking coffee and observing the Sámi artifacts on the walls, the knitted socks and mittens for sales and the old pictures probably of her parents and grandparents. After a while Bidos was served in a big pot”.

**Figure 4:**
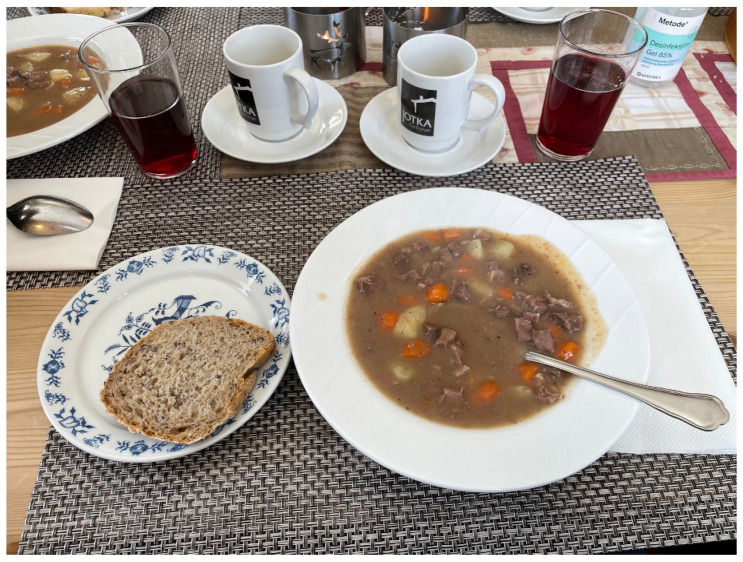
Bidos, a traditional Sámi soup made with reindeer meat, potatoes, and carrots, is often served during special occasions and festivals in Sámi culture.

“Bidos (see Figure 4) is a Sámi feast meal served at special occasions, like weddings, baptizing, confirmation etc. Bidos is reindeer in broth with potatoes and carrot. It was served with black current juice (saft). At weddings often 1000–2000 people were invited. The guest came when they had time. People eat at different times. Convenient with a big pot that was kept warm and where food could be added. As dessert, we got waffles with cloudberry jam, strawberry jam, sour cream and real goat cheese to add. Again, black coffee was served”.

The dimension of tradition vs. innovation captures the dichotomy between traditional and innovative sensory experiences. Traditional experiences emphasize culinary practices rooted in heritage and cultural norms, while innovative experiences involve novel approaches, fusion cuisine, or experimental techniques. The experiences can differ, reflecting the balance between familiarity and novelty. Two of the notes from the researchers illustrate this point:

“They explore the surroundings, try the tastes, and come up with novel ideas that, whilst maintaining the Sámi identity in the experience, push forward novel culinary experiences”.

“There seems to be a “tension” between traditional Sámi and the development of new Sámi ways. Informant 1 is sometimes criticized for not using all the Sámi imagery in the products and developments, yet Informant 1 considers themselves as developing the Sámi”.

Light vs. dark: The light vs. dark dimension relates to the contrasting experiences associated with brightness and darkness, which occur not only throughout the seasons (summer vs. winter) but also within the seasons. For example, in May, we observed an important contrast between how bright it was outdoors relative to indoors. One example of this is presented below, from the notes of one of the researchers:

“The experience starts outside. The tents, the Sámi symbolism, and the reindeer field sets up the stage. In this day of May, the snow and sun create a very bright atmosphere as well [which create a light contrast between indoors and outdoors] (…) The walls are covered with imagery of the previous location which visualizes the tents and the northern lights, as well as images of herds of reindeer. I did not perceive any particular smell but instead, the ample space appears to dissolve them. Once we sat down, in an area where there is a fireplace and cushions with colorful designs, we started talking. We had to change table, though, because the brightness of the outside atmosphere, entered through the window, and was blinding us”.

The dimension of culture vs. nature represents the interplay between cultural influences and natural elements in the experiences. Culture encompasses the traditions, customs, and practices that shape the experiences, while nature refers to the inherent qualities derived from the environment, ingredients, and geographical factors. Sensory attributes associated with culture may highlight specific flavors, spices, or cooking techniques, while natural elements may emphasize the intrinsic qualities of ingredients or the terroir of a particular region. This is captured, in part in the following field notes:

“Informant 2’s parents was Sámi from the East, the other from the West (without reindeer). Traditionally Sámi people lived on protein alone (fish in the river and reindeer). Potatoes introduced first in 1700. They ate everything (also the belly) of the reindeer and got the vitamin they needed from that”.

These bipolar dimensions provide a framework for understanding and describing the sensory experiences associated with different culinary traditions. By exploring the variations along these dimensions, researchers and chefs can gain insights into the cultural and regional influences on sensory perception and tailor their culinary creations accordingly.

*Sensory stories*. Sensory stories involve the narratives or storytelling elements associated with the eating experience [23,38]. They are used to enhance the overall sensory experience by providing a context or background information that enriches the perception and enjoyment of food or products. These stories can be personal anecdotes, cultural traditions, or historical accounts related to the sensory experience, and they tie together both the experience elements and the bipolar concepts into a broader story.

“…highlighted how all food comes with storytelling, which is key to the Sámi. Food experiences are not as such without the storytelling it appears. Informant 2 also highlighted the current social nature of the meal in the Sámi, where people gather around the dish, in this case Bidos, to eat and share together”.

“We did not taste anything (only coffee). But his stories made my mouth water. Sounds like he does everything right. He starts with the protein when planning a meal and gives the young chefs his ideas and let them elaborate on them (to add their own tough). They use seasonal raw material. Reindeer is used a lot. From shrimp leftovers he can make different ingredients (in milk, for fermentation (can be used for soup), in oil, powder”.

When a meal or a product is presented with a sensory story, it aims to evoke emotions, memories, and associations that enhance the consumer’s engagement and appreciation. The sensory story can set the stage, create a sense of anticipation, and guide the people’s attention toward specific sensory aspects of the experience. These narratives can foster emotional connections, cultural appreciation, and a deeper understanding of the culinary context.

*Values.* Values, in the context of the experiences documented, represent a series of principles or guiding beliefs that influence the formation and evaluation of experiences at a broader level. These values can shape individual preferences, decision making, and overall perceptions of sensory qualities. We identified four key values, namely, (1) sourcing locally, (2) sustainability, (3) solidarity, and (4) simplicity.

The value of *sourcing locally* represents a principle across the different businesses where experiences prioritized and emphasized locally sourced ingredients, products, or cultural elements.

“…food philosophy is to serve traditional Sámi food made from local raw material”.

“She likes to use the local available resource for her menus. No planning in advance. The menu depends on what she catches or finds during the day. It is spontaneous but it matches with nomadic spirit, appreciating the nature resources and make food accordingly to what is available”.

“The majority of these products are locally sourced and processed, with the theme of ‘Nordic Light’ prominently featured on their labels and packaging”.

The value of *sustainability*, as captured in the observations and interviews, encompasses principles that promote a relationship of balance between people and nature. 

A relevant observation is the adaptation of the culinary practices based on seasons and local availability. For instance, up in the highland, fish from a local lake is often served in the summer, while reindeer is served in the winter. They get whole reindeer delivered from the Sámi, pick berries, and catch fish. Hardly anything grows here, not even rhubarb, so carrots and potatoes are delivered from a local farmer down in the valley. One of the respondents explained how she really enjoyed starting the day without knowing what to serve the guests. Then she picked berries (blueberries or cloudberries) right outside the hut and caught fish from the local lake and turned this into a meal that she served the guests in the evening. She preferred to make all meals based on what nature and the season had to offer.

**Figure 5:**
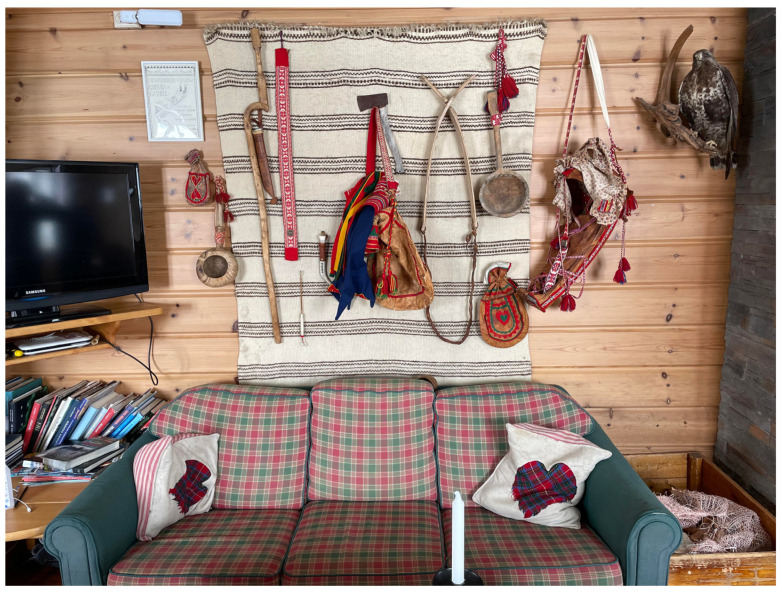
Sámi objects.

Interestingly, the traditional Sámi often practice a zero-waste approach, which means utilizing all the food resources. They use almost everything on the reindeer (tongue, heart, liver, blood, etc.), and traditionally, the gut was used to make a bag, while the fur become clothes and the antler turned into cutlery (see Figure 5 for examples of Sámi objects).

Also down in the valley, we observed similar sustainable practices. We noted how a modern gastronomy chef had developed great knowledge of utilizing the parts of plants and animals that people often overlooked. For instance, parts of the king crab not used by others were transformed into food, sap from the birch tree was served directly as drinks or boiled into syrup, and ice cream was made from bark. Interesting new flavors were composed by fermenting, boiling, salting, and drying almost everything. Fish skins were dried or put into the air-fryer to give them the right texture; berries were dried, put on water, frozen, and mixed in all kinds of ways. Even the ends of the leeks were cleaned and put into the air-fryer. We observed a sustainable, modern, and creative food philosophy utilizing everything the nearby nature had to offer. Here are some examples of our notes:

“Inspired by the surrounding nature, Informant 2 conceptualizes his dishes by envisioning the primary ingredient and then deliberates on its potential combinations and uses. His creativity shines through in his sustainable methods, such as crafting ice cream powder and sauces from pine trees. He emphasizes that an integral aspect of fine dining is not just the dish itself but the narrative behind it and the element of surprise. This surprise often emerges from his skillful use of ingredients and his unique methods of storage, fermentation, and food processing”.

The value of *solidarity* is also embedded within the Sámi food experiences. The chef mentioned the important role of food sources and that he often prefers meats from local hunters. A deep-rooted communal bond within Sámi community is also observed. The traditional feast “Bidos” further exemplifies this solidarity as it involves a large number of community members sharing food during important life events such as weddings or baptisms. Together, these evidenced the Sámi’s communal ties and traditions through their food experiences.

“Informant 2 highlighted the current social nature of the meal in the Sámi, where people gather around the dish, in this case Bidos, to eat and share together”.

“Informant 2, now a mentor, places great emphasis on recruiting and training local apprentices in his restaurant. He has a strong preference for meats procured from local hunters, as he believes that wild animals, often stress-free, produce tastier meats. For him, quality trumps price; he’s willing to pay whatever the hunter demands, showcasing the depth of trust and connection between them”.

The value of *simplicity* embodies a principle in which experiences emphasize clarity, minimalism, and a streamlined approach that minimizes complexity. Individuals who cherish simplicity seek out uncluttered and focused experiences that highlight essential sensory qualities. For instance, dietary habits of the Sámi and their traditional nomadic life is reflecting in their food philosophy: eat when hungry, eat fresh catch of the day, no particular future diet plan.

“The simple way to cook and serve is what define Sámi eating experience”.

“Minimalistic in a way, but consistently cozy. White plates with textured lines for the main, a more decorated plate for the bread, and later the waffles, and a glass for the drink. Other than the aromas of the Bidos, or later the jams, there’s not much more. The cutlery is metal-based and relatively simple”.

## 4. Discussion

The present research focused on studying the multisensory intricacies of food experiences in Northern Norway. By investigating a unique context with its specific environmental and cultural characteristics, we sought to expand our understanding of how contextual factors intertwine to shape food experiences. We suggest a contextual framework addressing four aspects of the context influencing food experiences in Northern Norway. The insights gained from this study will not only enrich our understanding of diverse food cultures but also inform the development of interventions and strategies aimed at enhancing sensory enjoyment. The multisensory food experiences in the northernmost region of Norway demonstrate a dynamic interplay among sensory elements and contextual factors such as cultural influences, seasonality, and weather conditions. Grounded in the research theory on multisensory food perception and experiences [1,7,39], these results contribute to a deeper comprehension of the multisensory food experience landscape and to a general framework that can be applied when investigating food experiences in WEIRD and otherwise unique contexts.

First, the outcomes highlight the profound connection between these multisensory food experiences and the elements derived from the natural environment. The study identifies three fundamental experience elements—plants, animals, and inanimate objects—that serve as foundational constituents shaping the sensory facets of these culinary encounters. Birch trees, cloudberries, reindeer, and the ubiquitous presence of wood are not merely ingredients but integral components contributing to flavors, aromas, textures, colors, shapes, and fragrances that collectively enrich the overall sensory milieu. These findings support the significance of sensory elements originating from the environment in shaping individuals’ perceptions of and satisfaction with the foods they consume [1,39]. Furthermore, the incorporation of sensory stories into the experiences, which narrate the contextual and historical dimensions of these elements, amplifies the emotional and cognitive connections between consumers and the culinary context, as supported by Mossberg and Eide [23].

Additionally, the bipolar dimensions—Sámi vs. Norwegian, tradition vs. innovation, light vs. dark, and culture vs. nature—exemplify the contrasts and diversity that characterize food experience encounters in this region. The tension between preserving Sámi authenticity while embracing broader Norwegian influences accentuates the intricate cultural interplay, an observation in line with the rich cultural heritage of the Sámi people [17]. Furthermore, the juxtaposition of traditional culinary practices rooted in heritage with innovative, experimental approaches support the role of these dimensions in differentiating and describing sensory experiences based on deeper, perhaps affective, meanings akin to those captured by the semantic differential technique [37].

Lastly, the results indicate the values that underpin the multisensory food experiences studied, providing a broader framework for their formation and evaluation. Four key values—focusing on sourcing locally, sustainability, solidarity, and simplicity—are discerned as guiding principles that shape individual preferences and perceptions. The emphasis on locally sourced ingredients and sustainable practices may be indicative, in part, of the environmental constraints and resource reliance prevalent in this region, as well as an important symbiosis with the context [13]. Furthermore, the communal bonds exemplified in traditions like “Bidos” and the value of simplicity align with the social and lifestyle facets deeply embedded in Sámi food experiences [21]. These findings reflect how these values contribute to the distinctive sensory arrangements and cultural significance of gastronomy in this region. 

### 4.1. Implications

This study presents and encourages research that might step away from a predominantly WEIRD perspective, offering fresh and diverse viewpoints of the multisensory eating experience from Indigenous groups with rich cultural heritages. Our findings show the depth of sensory experiences that are often overlooked by the mainstream research.

The proposed multisensory framework features the food experiences with major themes such as experience elements, bipolar concepts, sensory stories, and values. This can serve as a recommended guide for researchers who are interested in food experiences across cultures and traditions. Additionally, future research could benefit from incorporating interdisciplinary approaches to studying multisensory food experiences, as well as by comparing these experiences with those in other regions. This would help us understand the uniqueness and cultural significance of food experiences across regions and cultures.

Practitioners, including tour operators, policy makers in tourism, and private environmental and cultural organizations, can leverage our findings to offer authentic tourism experiences. For example, rather than merely serving guests with traditional meals, tour operators can enrich the experience by sharing the sensory narratives behind the dishes and their consumption contexts. Meanwhile, policymakers should engage with local communities when formulating sustainable strategies. Our study also highlights the value of adopting a no-waste approach concerning food resources and ingredients. Embedding with innovation and creative concepts, we can utilize the waste to create memorable experiences as well as to show respect to nature.

### 4.2. Limitations

One of the notable limitations of our study is its primary focus on the perspectives of a small group of business owners. This emphasis might not fully encapsulate the views and practices of daily Sámi consumers. Hence, while our findings offer valuable insights into the culinary traditions and sustainable practices from a business standpoint, they may not provide a comprehensive understanding of the broader Sámi community’s day-to-day culinary experiences and attitudes. Future research might target a larger sample size including a more diverse group of Sámi people. Notably, though, one of the advantages of the approach taken in the present research is the depth and holistic nature of the insights that can be obtained, which is a trade-off worth taking at times in studying topics like multisensory experiences in order to further contribute to our understanding of them. In the end, through utilizing multiple methods of inquiry, we might gain a better understanding of the phenomena being investigated.

Furthermore, while our research provides insights into the Sámi food experiences, our findings might not necessarily be applicable to other groups of people. We suggest future research be conducted in different contexts and diverse locations, where the voices and perspective of other groups, including Indigenous, should be studied.

We acknowledge the inherent subjectivity associated with field note-taking and observation methodologies in qualitative research. While we took deliberate steps to mitigate potential biases by maintaining the privacy of individual notes before group discussions, we recognize that personal perceptions and experiences inevitably influence the observational process. This recognition aligns with the principles of qualitative research, which value these nuanced insights. Future research might aim to replicate and extend the findings presented here, thereby enriching the understanding generated by our study.

## 5. Conclusions

Our study provides insight into the multisensory food experiences in the context of Northern Norway—a non-WEIRD viewpoint that has been overlooked in the mainstream studies. Our suggested framework would potentially benefit researchers and practitioners alike. The richness of our findings underscores the importance of focusing on the local, as well, in the context of multisensory food experiences. Indeed, future research should explore further the Indigenous practices and values through their unique multisensory food experiences for different, non-WEIRD demographic segments adopting and testing our framework. 

## Data Availability

The data presented in this study are available on request from the corresponding author due to restrictions on information regarding minority ethnic groups.

## Appendix A. Observation Guide

- Date and location
- General information about the observation setting: who does it (age, gender), where they do it, what they do
- Relationship to food in Norway/actor type
- Describe the eating experience(s), as detailed as possible.
- What food is eaten?
- Describe the multisensory elements of the experience: its look (sight), sound (hearing), smell, and feel (touch). Describe the elements of both the indoor and outdoor setting.
- How do the experience(s) change with the seasons? Inquire.
- What is eating/food philosophy?
- Notes and comments.

Guide for researchers:

*Aim.* To observe, experience, and document Sámi multisensory food experiences. The participant observation aim is directed toward the eating experiences and the extrinsic (not specific to the food itself, but everything else) multisensory components of them, that is, what they look (sight), sound (hearing), smell (ortho-nasal olfaction), and feel (touch) like. We may also document other sensory properties, such as whether people eat standing up or sitting (e.g., vestibular sensations). In addition, we will document the dishes that are part of said experiences. We should both observe but also experience, from a first-person perspective, each of the experiences.
